# Targeting CD5 chimeric antigen receptor-engineered natural killer cells against T-cell malignancies

**DOI:** 10.1186/s40164-024-00577-5

**Published:** 2024-10-26

**Authors:** Yingling Zu, Quan Ren, Jishuai Zhang, Hongchang Su, Qiumei Lu, Yongping Song, Jian Zhou

**Affiliations:** 1 Department of Hematology, The Affiliated Cancer Hospital of Zhengzhou University & Henan Cancer Hospital, Zhengzhou, Henan 450008 China; 2 Department of Pediatrics, The First Affiliated Hospital of Zhengzhou University, Zhengzhou, Henan 450052 China; 3 Shenzhen Pregene Biopharma Company, Ltd, Shenzhen, Guangdong 518118 China; 4 Department of Hematology, The First Affiliated Hospital of Zhengzhou University, Zhengzhou, Henan 450052 China

**Keywords:** Chimeric antigen receptor, Nature killer cells, CD5, T-cell malignancies

## Abstract

**Background:**

Chimeric antigen receptor engineered T cells (CAR-T) have demonstrated promising clinical efficacy in B-cell malignancies, and the approach has been extended to T-cell malignancies. However, the use of allogeneic T cells in CAR therapy poses a challenge due to the risk of graft-versus-host disease. Recently, natural killer (NK) cells have exhibited “off‑the‑shelf” availability. The nanobody-based CAR structures have attracted much attention for their therapeutic potential owing to the advantages of nanobody, including small size, optimal stability, high affinity and manufacturing feasibility. CD5, a common surface marker of malignant T cells, has three scavenger receptor cysteine-rich domains (D1-D3) in the extracellular region. The present study aims to construct “off‑the‑shelf” CAR-NK cells targeting the membrane-proximal domain of CD5 derived from nanobody against T-cell malignancies.

**Methods:**

Anti-CD5-D3 nanobody was screened by phage display technology, followed by constructing fourth-generation CAR plasmids ectopically producing IL-15 to generate CD5 CAR-NK cells derived from peripheral blood. And the second-generation CD5 CAR-T cells based on nanobody were generated, referred to as 5D.b CAR-T and 12 C.b CAR-T. Furthermore, CAR-NK cells without IL-15 (IL-15^△^ CAR-NK) were generated to assess the impact on cytotoxicity of CAR-NK cells. Cytotoxic activity against CD5^+^ hematologic malignant cell lines and normal T cells was exerted in vitro and NOD/ShiLtJGpt-Prkdcem26Cd52Il2rgem26Cd22/Gpt mouse model transplanted with Jurkat-Luc cells was used to evaluate the antitumor efficacy of CD5 CAR-NK cells in vivo.

**Results:**

Two nanobodies (5D and 12 C) competed for binding to the epitope of CD5-D3. 12 C CAR-NK cells were superior to 5D CAR-NK cells in antitumor potential and 12 C.b CAR-T cells exhibited superior cytotoxic activity than 5D CAR-T cells ex vivo. So, 12 C was regarded as the optimal nanobody. 12 C CAR-NK cells and IL-15^△^ CAR-NK cells exhibited robust cytotoxicity against CD5^+^ malignant cell lines and controlled disease progression in xenograft mouse model. 12 C CAR-NK cells demonstrated greater antitumor activity compared to that of IL-15^△^ CAR-NK cells in vitro and in vivo.

**Conclusions:**

Taken together, the fourth-generation nanobody-derived anti-CD5 CAR-NK cells may be a promising therapeutic against T-cell malignancies.

**Supplementary Information:**

The online version contains supplementary material available at 10.1186/s40164-024-00577-5.

## Introduction

T-cell malignancies represent a class of extensively heterogeneous diseases with high rates of relapse and mortality. T-cell acute lymphoblastic leukemia (T-ALL) accounts for approximately 15% of childhood and 25% of adult ALL, respectively [1]. T-lymphoblastic lymphoma encompasses biological properties akin to T-ALL. Patients with T-cell malignancies tend to have a dismal prognosis [2, 3], absence of curative or targeted therapies beyond hematopoietic stem cell transplantation (HSCT) accompanied by potential serious toxicity and complications. Hence, it highlights the need for innovative therapeutics.

CD5 is highly expressed in T-ALL and T-cell lymphoma in addition to a fraction of B cell malignancies [4–6], such as mantle cell lymphoma (MCL) and chronic lymphocytic leukemia (CLL), rather than hematopoietic stem cells and natural killer (NK) cells [7]. As a negative regulator of T-cell receptor (TCR) signaling [8], CD5 is a potential target antigen for chimeric antigen receptor (CAR) against T-cell malignancies and had been validated in previous clinical trials using CD5 CAR-modified T (CAR-T) cells [9–11]. CD5 is a 67-kDa type I glycoprotein, including both transmembrane and secreted proteins [12, 13]. The extracellular region of CD5 is composed of three scavenger receptor cysteine-rich (SRCR) domains (D1, D2, and D3) [14–16]. Several preclinical studies of antibody-derived CAR-T cell therapies showed that binding membrane-proximal epitope had been revealed to enhance the immune effector function [17–20]. Whereas, approaches for CAR-T cells against T-cell malignancies remain a major impediment. These challenges could be attributed to the fratricide of CAR-T cells expressing shared phenotype with targeted malignant cells [21], and circulating malignant T cells probably existing in CAR-T cells derived from autologous T cells [22, 23] as well as a risk of graft-versus-host-disease (GVHD) due to allogeneic T cells as the source of CAR-T cells.

NK cell, a crucial part of the innate immune system, is a promising strategy for CAR engineering. NK cells have been utilized as effector cells without dependent on major histocompatibility complex (MHC), accompanied by limited risk of GVHD and cytokine release syndrome (CRS) [24]. Moreover, engineered NK cells mediated potential cytotoxicity relied on the presence of a full array of native receptors other than specificity of the CAR, avoiding the relapse incurred by the loss of CAR-targeted antigen [25, 26]. Therefore, CD5 CAR NK-92 cells exhibited viable strategy for T-cell malignancies [9, 27]. Nevertheless, NK-92 cells require irradiation prior to clinical application resulting in the short lifespan in vivo. An alternative approach employs CAR modified primary NK cells to address potential concerns. With regard to survival and proliferation in vivo, engineered anti-CD19 CAR-NK cells with IL-15 and inducible caspase-9 had successfully entered phase II clinical trial (NCT03056339) and the overall response rate (ORR) was up to 73% (8/11) without obvious toxic effects [28].

Nanobody (VHH) is derived from the variable domain of heavy-chain antibodies of.

Camelidae family. Compared to single-chain variable fragment (scFv), the distinct advantage of VHH is low immunogenicity apart from high affinity and specificity [29, 30]. Herein, we screened out two VHHs specially identified membrane-proximal domain (D3) of CD5 (CD5-Trunc). For the first time, an “off-the-shelf” strategy that anti-CD5-Trunc VHH-based CAR-NK cells derived from peripheral blood mononuclear cells (PBMCs) to ectopically produce IL-15 was used to treat T-cell malignancies. We successfully established the manufacturing and expansion protocols of primary CAR-NK cells. It was demonstrated that CD5 CAR-NK cells exhibited rapid proliferation and potent cytolysis against malignant T cell lines. Furthermore, the outstanding cytotoxicity of CAR-NK cells was shown in xenograft mouse models.

## Methods

### Cells and culture conditions

Peripheral blood (PB) from healthy donors was obtained from the Affiliated tumor hospital of Zhengzhou university and approved by the Ethics Committee of the Affiliated tumor hospital of Zhengzhou university. SUPT-1 and 293T cell lines were purchased from ATCC (Manassas, VA, USA), and Jurkat cell line was purchased from Guangzhou Cellcook Biotech Co.,Ltd. CCRF-CEM, Jeko-1 and Raji cell lines were purchased from Institute of Basic Medicine, Chinese Academy of Medical Sciences (Beijing, China). All tumor cell lines were incubated in RPMI medium (Hyclone, Logan, UT, USA) supplemented with 10% fetal bovine serum (FBS) (Gibco, Grand Island, NY, USA). NK cells derived from PBMCs were purified via CD3^+^ cells depletion followed by CD56^+^ cells enrichment, and maintained in NK MACS Medium (NK basal medium (Miltenyi Biotec, Bergisch Gladbach, Germany) supplemented with 1% of NK MACS Supplement, 5% CTS Immune Cell Serum, and combination of 500 IU/mL IL-2 (SI HUAN SHENG WU, Beijing, China) and 28 ng/mL IL-15 (Miltenyi)). Another culture method, engineered K562 feeder cells expressing membrane-bound IL-21 and CD137 ligand combined with exogenous IL-2 (200 IU/mL), was also adopted. Primary T cells were positively isolated from PBMCs using CD3 microbeads (Miltenyi). CAR-T cells were cultured in TexMACS GMP Medium (Miltenyi) supplemented with 100 IU/mL IL-2 and 5% CTS Immune Cell Serum Replacement (Gibco), activated with 1% TransAct reagent (Miltenyi). 293TS cells were incubated in 50% SMM 293-TII, 50% OPM-293 CD05 Medium (OPM) and 6mM L-glutamine (Sigma-Aldrich, St. Louis, MO, USA), and 293T cells were maintained in DMEM Medium (GE Healthcare life sciences) supplemented with 10% FBS (Gibco). Jurkat cells were transfected with Luciferase lentivirus to establish the Jurkat-Luciferase (Jurkat-Luc) cell line. CD5-Trunc expression vector was constructed, and Raji cells were transfected with the vector for establishment of stable CD5-Trunc overexpressed Raji (CD5^+^ Raji) cell line.

### Immunization and library construction

An adult, healthy alpaca was immunized with CD5-His protein coupled with Freund’s adjuvant (Sigma-Aldrich) in two weeks interval. After the fourth immunization, serum was separated to monitor the antibody responses by ELISA. Peripheral blood lymphocytes (PBLs) were separated by density gradient centrifugation to extract total RNA. The phage display library was constructed according to mRNA as a template for reverse transcription polymerase chain reaction (RT-PCR), amplifying the VHH encoding sequences and ligating VHH repertoire into phagemid pComb3XSS. The capacity of VHH library performing in *E.coli* TG1 strain was calculated by counting the numbers of colony after gradient dilution. The correct insertion rate and diversity of constructed library were displayed by PCR amplification and gene sequencing, respectively.

### VHH selection by phage display, expression, purification and identification

To obtain CD5-Trunc protein for VHH selection, CD5-Trunc-Fc plasmid was transiently transformed into 293T cells for eukaryotic expression. FC-conjugated CD5-Trunc protein was purified by protein A affinity chromatography (GE Healthcare). Commercial His-conjugated CD5-full length (CD5-FL) protein and FC-conjugated CD5-Trunc protein were coated for biopanning, respectively. After four rounds of panning, CD5-positive clones were sequenced. Clones with different complementarity determining region containing 6×His tag were subcloned into PSJF2 vector, which were transformed into *E.Coli* TG1 strain for expression of VHH. For eukaryotic expression, Fc-tagged VHH plasmids were transfected into 293TS cells. The FC-tagged VHHs were purified by protein A affinity chromatography (GE Healthcare), and 6×His-tagged VHHs were purified through affinity chromatography with Ni NTA Beads (Smart-Lifesciences, Changzhou, China). The purity of VHHs was determined using sodium dodecyl sulfate-polyacrylamide gel electrophoresis (SDS-PAGE). Jurkat, Raji and 293T cells over-expressed CD5-FL and CD5-Trunc were used to evaluate binding specificity of CD5 VHH antibody with endogenous and exogenous expression of cell surface molecules. Indirect-ELISA assay was performed to validate binding specificity indirectly and species reactivity of VHH. Concentration for 50% of maximal effect (EC_50_) was obtained after incubation of Jurkat cells and different gradient VHHs using flow cytometry. Binding competition study by flow cytometry was assayed the binding epitope of each VHH on target antigen.

### CD5-CAR construct design and transduction

To construct CAR-T targeting CD5, the anti-CD5 VHH was linked to CD8α signal peptide, CD8α hinge and transmembrane, and the intracellular domain of 4-1BB and CD3ζ of an anti-CD5-4-1BB-CD3ζ CAR. To avert the fratricide of CD5 CAR-T attributing to surface marker profile largely overlapped with surface antigen of malignant T cells, the same VHH sequence was joined to the sequences encoding endoplasmic reticulum (ER) retention peptides, coupled with the second-generation CD5 CAR mentioned above. As for CAR-NK, the anti-CD5 VHH was cloned into the backbone of a fourth-generation CAR with CD28-derived hinge, transmembrane and intracellular domain fused to CD3ζ signaling domain, coupled with the human IL-15 gene via a T2A peptide. Besides, the fourth-generation CAR lacking the human IL-15 gene was constructed to investigate the potential of IL-15 to effect on CAR-NK cytotoxicity.

The generated CD5-CAR cassettes were subcloned into BamHI and SalI sites of the Pre-Lenti-EF1-CAR V2 WPmut vector, as the CD5-CAR plasmid. The lentiviral supernatant containing CD5-CAR for NK cells was produced by 293TS cells transfected with CD5-CAR lentiviral vector and three packaging plasmids, BaEVRLess, PMDLG and pRSV [31, 32], and the lentiviral supernatant for T cells was also produced by 293TS cells transfected with CD5-CAR lentiviral vector and the packaging plasmids, PMDLG, PMD2G and pRSV. Viral supernatant was harvested 48 h after transfection, and then filtered and concentrated by ultracentrifugation. NK cells were suspended at 1 × 10^6^ cells/mL in 400µL expansion NK MACS Medium containing 10 µg/mL Vectofusin-1 and supplemented with lentiviral supernatant at a multiplicities of infection (MOI) of 10 on day 3 based on stimulation with cytokines alone or on day 6 stimulated with cytokine in combination with engineered K562 feeder cells. T cells were transfected with lentiviral supernatant at a MOI of 3 and supplemented with 1 µg/mL polybrene on day 2.

### Flow cytometry

PE-conjugated CD56 and FITC-conjugated CD3 antibodies were used to label primary NK cells and T cells. FITC-conjugated CD56 and PE-conjugated CD5 antibodies were exploited to test the CD5 expression on the surface of NK cells. PE-conjugated anti-CD5 antibody was applied to detect the CD5 expression on the surface of matural T cells and tumor cell lines. All the antibodies were purchased from Biolegend, lnc. NK cells and T cells were incubated with PE-Labeled Human CD5 protein, His Tag (ACROBiosystems, China) for CD5-CAR expression. The data was analyzed with FlowJo 7.6.

### Proliferation and cytotoxicity assay

To evaluate proliferative effect on account of IL-15, we compared the proliferation of NK supplemented with 500 IU/mL IL-2 and 28 ng/mL IL-15 to that of NK only supplemented with 500 IU/mL IL-2. NK cell viability with different concentrations of IL-15 was evaluated to explore optimal concentration of IL-15, averting NK cell addiction to IL-15. Cells were counted via trypan blue staining every other day. The target cells, including Jurkat, CCRF-CEM, Jeko-1, SUP-T1 and CD5^+^ Raji, were used as CD5^+^ cells and Raji cells were served as CD5^−^ target cells. The normal T cells were used to evaluate the side effects of CAR-NK cells. The effector cells were co-cultured with target cells at various effector-to-target (E: T) ratios overnight. The cytotoxicity of CAR-NK cells to CD5^+^ tumor cell lines was assessed via LDH cytotoxicity assay (promega, Wisconsin, USA). All experiments were carried out in triplicate.

### Cytokine secretion and RNA sequencing

CD5 CAR-T cells and nontransduced T (NT-T) cells were incubated with target cells at ratio 1:2, 1:4 and 1:8 overnight. CD5 CAR-NK cells and nontransduced NK (NT-NK) cells were performed with target cells and at various ratios overnight. Following co-culture, supernatant was harvested to evaluate the production of IFN-γ, IL-15 and Granzyme B using ELISA kits (IFN-γ: BioLegend, San Diego, CA, USA; IL-15: R&D Systems, Minneapolis, MN, USA; Granzyme B: solarbio, Beijing, China) according to manufacturer’s instructions.

### Mouse xenograft model

Six- to eight-week-old female NCG mice (NOD/ShiLtJGpt-*Prkdc*^em26Cd52^*Il2rg*^em26Cd22^ /Gpt) purchased from GemPharmatech Co.,Ltd (Nanjing, China) were intravenously injected with 3 × 10^6^ Jurkat-Luc cells. Mice were randomly divided into four treatment groups: group freezing solution (75% CS10 + 25% HSA, control), group frozen Mock-NK, group frozen 12 C CAR-NK and group frozen IL-15^△^ CAR-NK, all cells were cultured for two days under CAR-NK condition postthawed. Eleven days after tumor engraftment, a single of 5 × 10^6^ CAR-NK cells or NK cells was injected into each mouse intravenously. At days 3, 7, 14, 21, 24 and 31, bioluminescence imaging (BLI) was performed to monitor tumor burden. Mice were euthanized after four weeks or reaching the standard of euthanasia in the solvent control group. All procedures were in compliance with the regulations of experimental animal management committee of the Affiliated tumor hospital of Zhengzhou university.

### Statistical analysis

Unpaired student t-test was used to compare quantitative differences between two samples, and two-way ANOVA was applied for multiple comparisons. Data were expressed as mean ± standard deviation (SD). *p* < 0.05 was considered statistically significant and *p* values were two-sided. The significances were indicated as * for *p* < 0.05, ** for *p* < 0.01, *** for *p* < 0.001 and **** for *p* < 0.0001. Survival curves were performed using Kaplan–Meier method and differences in statistical analyses were compared with the log-rank test. All statistical analyses were performed in GraphPad Prism 7.0 software.

## Results

### Discovery and identification of anti-CD5 VHH

**Fig. 1:**
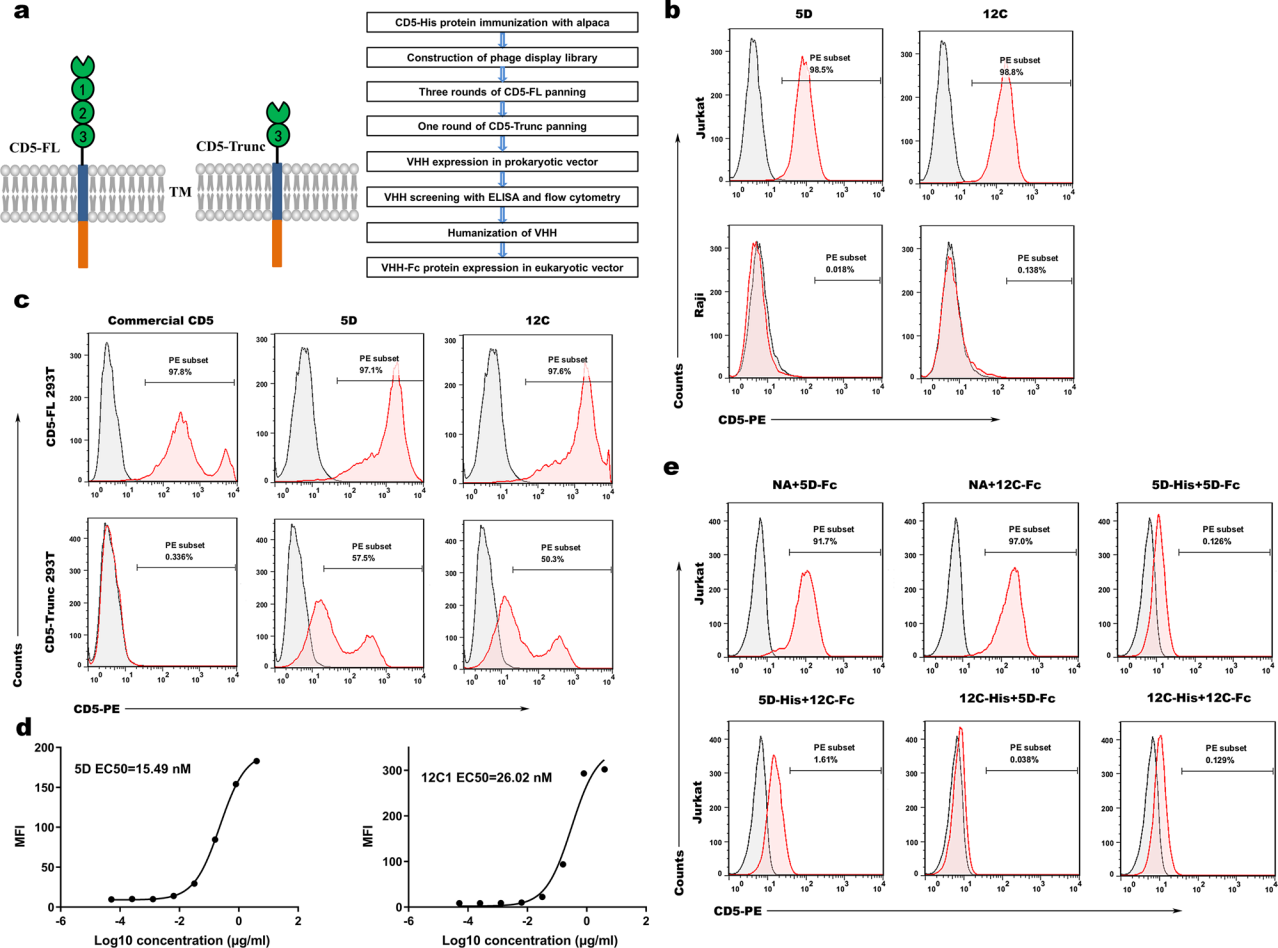
Discovery and identification of anti-CD5 VHH. (**a**) Structure chart of CD5-FL and CD5-Trunc protein (left panel) and flow chart of anti-CD5 VHH discovery process (right panel). (**b**) Two selected VHHs (5D and 12 C) specifically recognized CD5^+^ Jurkat cells but not CD5^-^ Raji cells. (**c**) The ability of two VHHs to specifically recognize exogenously expressed cell surface molecules. 5D and 12 C specifically bound to CD5-FL overexpressed 293T (CD5-FL 293T) cells and CD5-Trunc overexpressed 293T (CD5-Trunc 293T) cells. Commercial CD5 antibody specifically recognized CD5-FL 293T cells instead of CD5-Trunc 293T cells. (**d**) The binding affinity of 5D and 12 C on the Jurkat cell membrane. 5D-Fc and 12 C-Fc were administrated to test the binding ability using flow cytometry. (**e**) 5D and 12 C competed for binding to the epitope of CD5 molecule on the Jurkat cell membrane, as determined with binding competition studies by flow cytometry. Jurkat cells were incubated away from light with blank control, 5D-His and 12 C-His at 4℃ for 30 min, two of each group and a total of six groups. After two washes with PBS, 5D-Fc and 12 C-Fc were added into samples successively. After incubation again, PE anti-human IgG Fc secondary antibody was added, followed by sample examination by flow cytometry. CD5-FL: Full-length CD5; CD5-Trunc: Membrane-proximal domain of CD5; VHH: Variable domain of heavy chain of heavy-chain

Total RNA, extracted from PBLs of alpaca immunized with CD5-His protein, was reverse transcribed to cDNA as the template for PCR. A phage antibody library was constructed after double digests, connection and conversion. CD5 VHH library was enriched via three rounds of CD5-FL protein and one round of CD5-Trunc protein biopanning (Fig. 1a). Afterwards, 8 CD5-positive clones of 92 clones were generated via indirect-ELISA and sequenced. The humanized VHH fragments were subcloned into *E*. coli TG1 strains, and tagged with 6xHis tag for purification. After expression and purification, two VHHs (5D and 12 C) were demonstrated specific binding ability with CD5^+^ Jurkat cells but not CD5^−^ Raji cells (Fig. 1b). Two VHHs electrophoresed in SDS-PAGE had single band with high purity (Fig.S1a). To investigate the binding specificity of VHHs, we incubated VHHs with CD5-FL and CD5-Trunc transient line 293T cells (CD5-FL 293T and CD5-Trunc 293T). It was exhibited that 5D and 12 C specifically bound to CD5-FL 293T cells and CD5-Trunc 293T cells. In contrast, commercial CD5 antibody specifically bound to CD5-FL 293T cells instead of CD5-Trunc 293T cells (Fig. 1c). We labeled Jurkat cells with diluted CD5-Trunc-Fc protein and calculated the EC_50_ of 5D and 12 C as 15.49nM and 26.02nM, respectively (Fig. 1d). Moreover, both VHHs with 6×His tag or Fc tag competed with each other for binding to Jurkat cells by flow cytometry (Fig. 1e). Binding competition studies demonstrated that 5D and 12 C competed for binding to the epitope of CD5 molecule. On account of differences in amino acid sequence of D3 between the human and mouse CD5 protein (Fig.S1b), species specificity assay revealed that two VHHs bound to CD5-FL and CD5-Trunc proteins with high-binding ability, but not to mouse CD5 protein. Optical density (OD) at 450 nm was depicted in Fig.S1c.

### Blocking the expression of CD5 prevents T-cell fratricide and 12 C CAR-T cells display superior cytotoxicity against CD5+ hematologic malignant cells in vitro

**Fig. 2:**
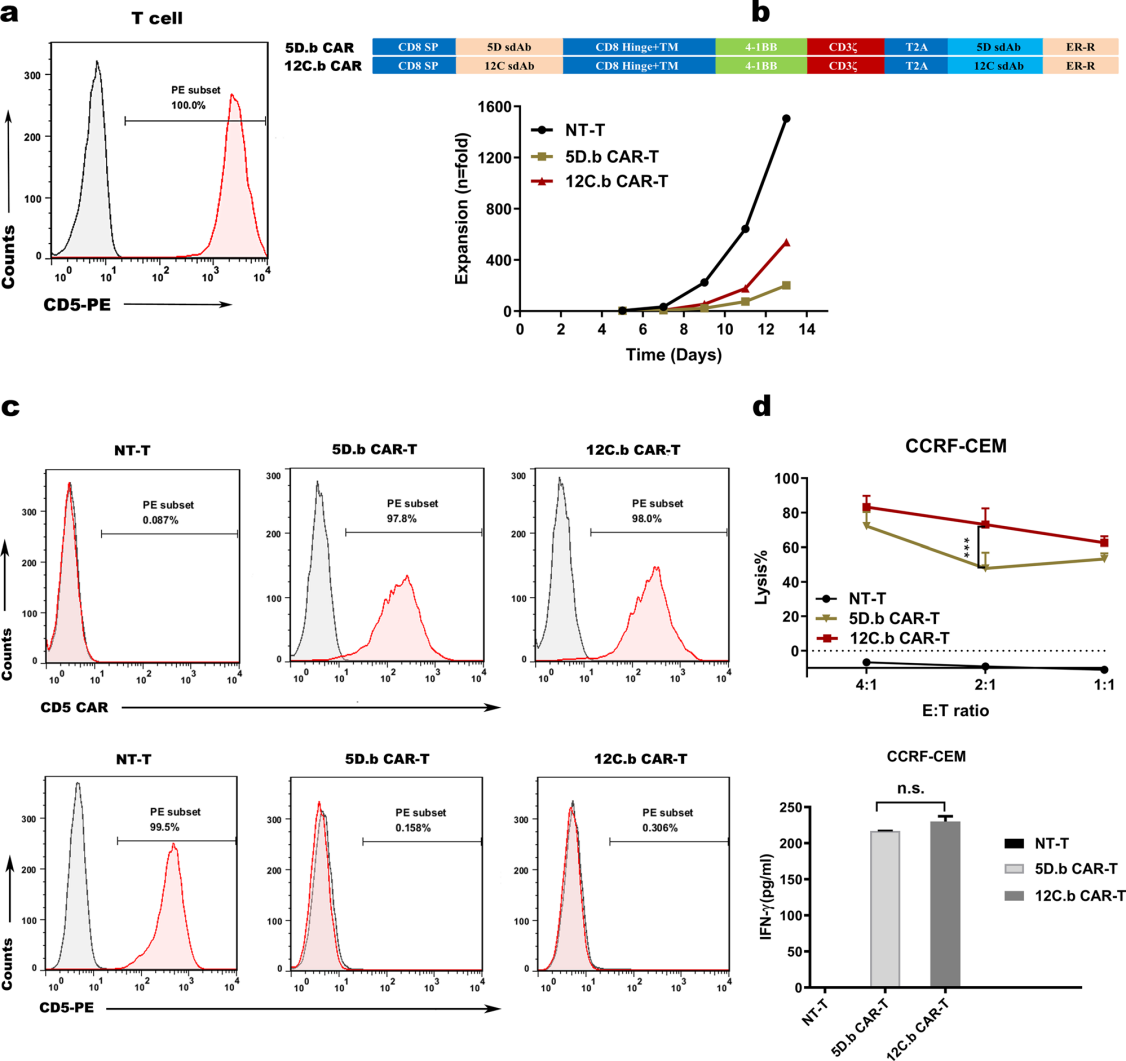
Preparation of CD5 CAR-T cells and 12 C.b CAR-T cells display superior cytotoxic activity against CD5^+^ hematologic malignant cells in vitro. (**a**) Expression of CD5 in normal matural T cell. (**b**) Schematic diagram of the two CD5 CAR lentiviral vectors (top panel) and expansion of T cells transduced with CD5 CARs in vitro for 13 days (bottom panel). (**c**) Surface expression of the CD5 CAR constructs (top panel) and CD5 molecule (bottom panel) on transduced T cells measured by flow cytometry on day 12. (**d**) Cytotoxicity of CD5 CAR-T cells against CD5^+^ CCRF-CEM cells. CD5^+^ CCRF-CEM cells and CD5 CAR-T cells or NT-T cells were cocultured overnight at the indicated E: T ratio (*n* = 3; two-way ANOVA; ***p < 0.001) (top panel). LDH assay showing the lysis of target cells. ELISA data showing the release of IFN-γ by NT-T cells and CD5 CAR-T cells after co-incubation with target cells overnight (*n* = 3; n.s., no significance) (bottom panel). NT-T: nontransduced T

To compare the effects of VHH 5D and 12 C, we constructed an anti-CD5-4-1BB-CD3ζ CAR that contained CD8α hinge and transmembrane, as well as the intracellular domain of 4-1BB in tandem with CD3ζ. We then transduced the CAR vector into human primary T cells. Unfortunately, the T cells expressing anti-CD5-4-1BB-CD3ζ CAR failed to expand compared to the control T cells (data not shown). The impaired growth of CAR-T cells was attributed to unintended fratricide, which mediated by the CAR binding to CD5 expressed by the T cells (Fig. 2a). To address the drawback, T cells were transduced with the second-generation CD5 CAR mentioned above which was linked to amino acid sequences encoding ER retention, referred to as 5D.b CAR-T and 12 C.b CAR-T (Fig. 2b). Membrane expression or secretion of the targeted protein was blocked by fastening the construct to the ER [33, 34]. After 12 days of culture, we observed normal expansion of 5D.b CAR-T and 12 C.b CAR-T cells (Fig. 2b). As expected, CD5 surface expression in CAR-T cells was essentially abrogated, and flow cytometry showed that CD5 CARs expressed on T cells were nearly 100% on day 12. (Fig. 2c). As two VHHs (5D and 12 C) competed for binding to the epitope of CD5 protein, we selected the optimal VHH by evaluating the cytotoxic effect of 5D.b CAR-T and 12 C.b CAR-T. After coculture with CD5^+^ CCRF-CEM cells overnight (Fig.S2a), 12 C.b CAR-T cells exhibited superior cytotoxic activity than 5D CAR-T cells (Fig. 2d). Furthermore, IFN-γ, a vital cytokine linking to activation and cytotoxic activity of T cells functionally [35], was detected. But, the data showed that the cytokine released by 12 C CAR-T cells was comparable to that released by 5D CAR-T cells when cocultured with CD5^+^ hematologic malignant cells (Fig. 2d). The results indicated that abrogation of CD5 expression in T cells averted the self-antigen-driven fratricide and 12 C CAR-T cells exhibited higher cytotoxicity efficacy towards CD5^+^ CCRF-CEM cells than 5D CAR-T cells in vitro.

### NK cells derived from PB expand ex vivo and 12 C CAR-NK cells exhibit superior cytotoxic activity

**Fig. 3:**
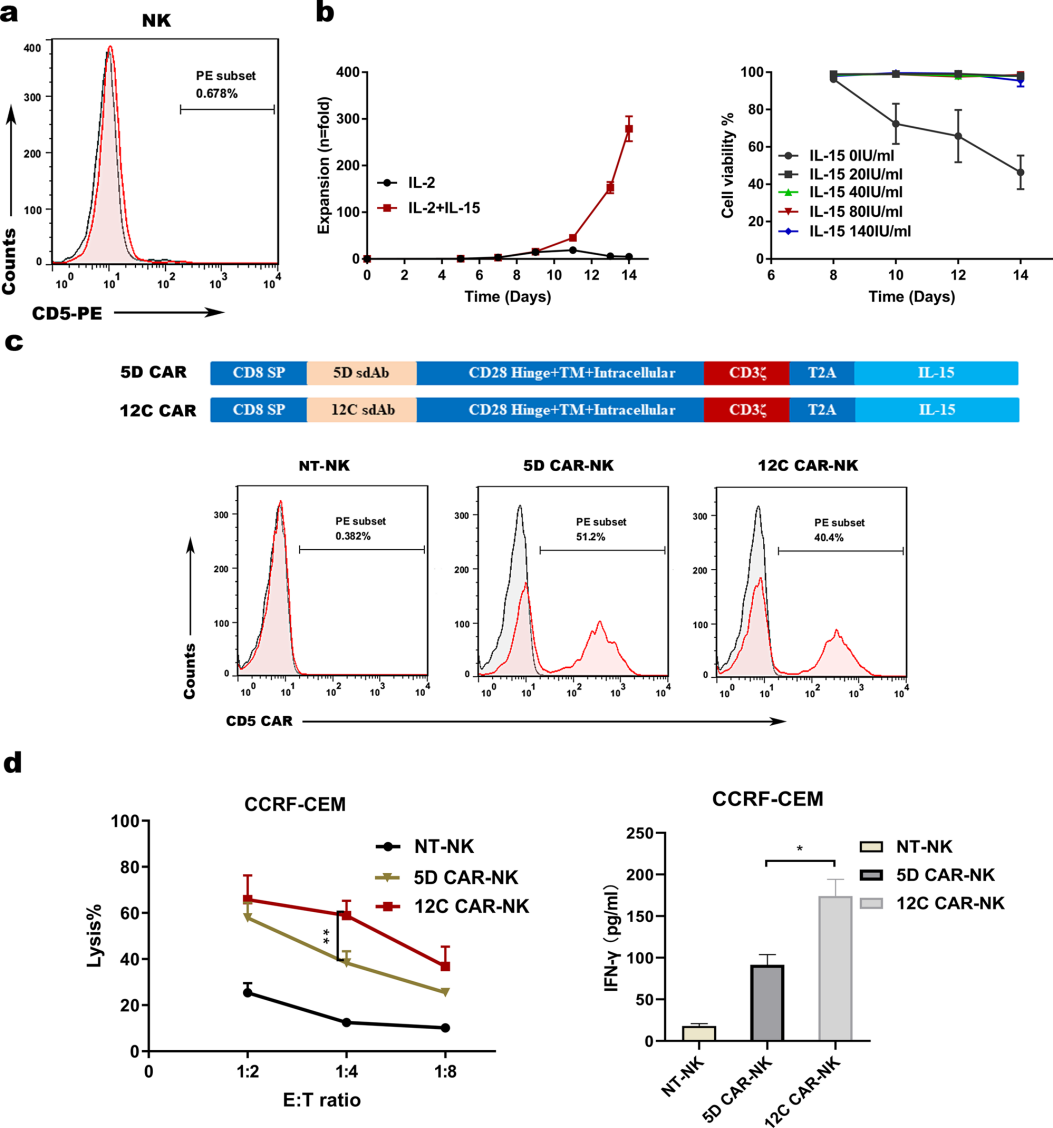
Preparation of CD5 CAR-NK cells with cytokine stimulation and 12 C CAR-NK cells exhibit predominant anti-CD5^+^ hematologic malignant cell activity ex vivo. (**a**) Flow cytometry analysis showing no expression of CD5 on peripheral blood NK cells. (**b**) Expansion of NK cells with IL-2 combined with IL-15 or without IL-15 for 14 days (*n* = 3, left panel), and cell viability of NK cells with different concentrations of IL-15 (*n* = 3, right panel). (**c**) Schematic diagram of the two lentiviral CD5 CAR expression plasmids (top panel) and flow cytometry analysis of CD5 CAR expression on CAR-NK cells versus NT-NK cells on day 14 (bottom panel). (**d**) Cytotoxicity of CD5 CAR-NK cells against CD5^+^ CCRF-CEM cells. CD5^+^ CCRF-CEM cells and CD5 CAR-NK cells or NT-NK cells were cocultured overnight at the indicated E: T ratio (*n* = 3; two-way ANOVA; **p < 0.01) (left panel). LDH assay showing the lysis of target cells. ELISA data showing the release of IFN-γ by NT-NK cells and CD5 CAR-NK cells after co-incubation with target cells overnight (*n* = 3; *p < 0.05) (right panel). NT-NK: nontransduced NK

Contrary to T cells, NK cells derived from PB possess nearly no expression of CD5 (Fig. 3a). To observe the contribution of IL-15 to NK cells proliferation in the way of cytokine stimulation, it displayed that the expansion of NK cells with 500 IU/mL IL-2 and 28 ng/mL IL-15 (279 fold) was obviously superior to that of NK cells without IL-15 (4.77 fold) after culture for 14 days (Fig. 3b), demonstrating that IL-15 was a indispensable cytokine for the growth of NK cells. Furthermore, we optimized the culture condition of NK cells through evaluating the effect of different IL-15 concentrations on the proliferation of NK cells. The results showed that different IL-15 concentrations had no effect on the viability of NK cells except for the concentration of 0 ng/mL (Fig. 3b). In brief, IL-15 is an essential cytokine for NK cell development and survival.

In order to generate CD5 CAR-NK cells, two CAR vectors with different VHHs (5D and 12 C) were constructed, consisting of CD28-derived hinge, transmembrane and intracellular domain coupled with CD3ζ signaling domain, and the human IL-15 gene via a T2A peptide (Fig. 3c). NK cells derived from PB were infected with CAR structure lentivirus to generate 5D CAR-NK cells and 12 C CAR-NK cells, resulting in above 40% of CAR expression (51.2% for 5D CAR-NK and 40.4% for 12 C CAR-NK, respectively) on day 14 (Fig. 3c). To further verify the cytotoxicity of CAR-NK cells, CCRF-CEM cells were selected as the positive target cells. After coculture with CCRF-CEM cells overnight, 5D CAR-NK cells and 12 C CAR-NK cells exerted robust and specific tumor-killing activity relative to control NK cells, and stronger cytotoxicity and more IFN-γ release were observed in 12 C CAR-NK cells compared to those in 5D CAR-NK cells (Fig. 3d). These data further strengthened that 12 C was the optimal VHH antibody and 12 C CAR-NK cells exhibited greater cytotoxic activity against CD5^+^ CCRF-CEM cells than 5D CAR-NK cells ex vivo.

### 12 C CAR-NK cells effectively recognize and eliminate CD5+ hematologic malignant cells and normal T cells

**Fig. 4:**
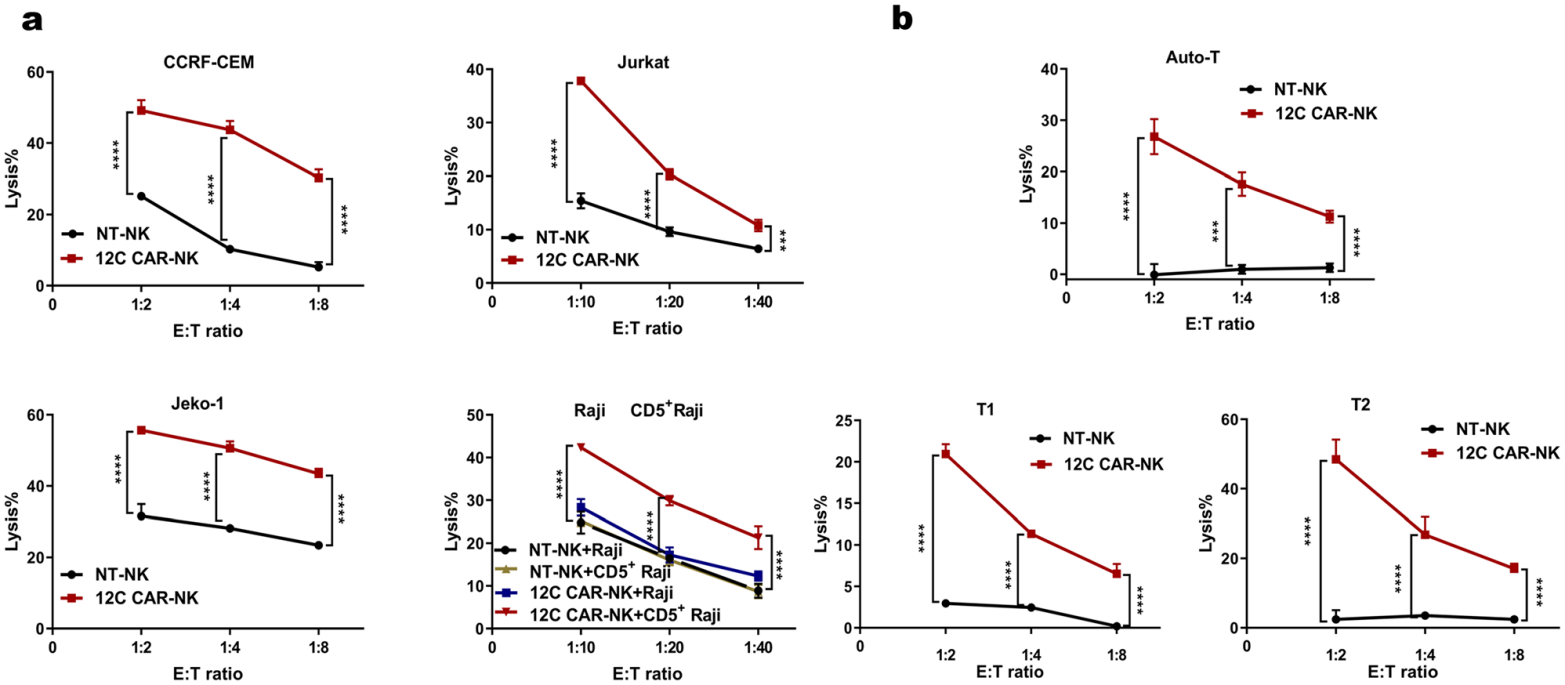
12 C CAR-NK cells with cytokine stimulation specially recognize and kill CD5^+^ hematologic malignant cells and normal T cells in vitro. (**a**) Cytotoxicity of 12 C CAR-NK cells and NT-NK cells cultured with cytokine stimulation, IL-2 combined with IL-15, against CD5^+^ malignant tumor cell lines was assessed. CD5^−^ Raji cells were used as a negative control. Effector cells were incubated with target cells overnight at the indicated E: T ratio (*n* = 3; two-way ANOVA; ***p < 0.001, ****p < 0.0001). (**b**) Cytotoxicity of 12 C CAR-NK cells and NT-NK cells against normal T cells. 12 C CAR-NK cells and NT-NK cells were cocultured with auto normal T cells (top panel) and two donors’ normal T cells (T1, T2; bottom panels) overnight at the indicated E: T ratio (*n* = 3; two-way ANOVA; ***p < 0.001, ****p < 0.0001). NT-NK: nontransduced NK; CD5^+^ Raji: Raji overexpressed membrane-proximal domain of CD5; Auto: Autologous; T1 and T2: Two donors’ normal T cells

To further evaluate the cytotoxicity of 12 C CAR-NK cells cultured with cytokine stimulation, four different target cells were selected. Raji cell line was used as negative control without expression of CD5, while the cell lines of T cell leukemia Jurkat and CCRF-CEM, the cell lines of MCL Jeko-1 and CD5^+^ Raji were used as target cell lines (Fig.S2a-c). After overnight coculture with target cells at different E: T ratios, relative to spontaneous cytotoxicity of NT-NK cells, 12 C CAR-NK cells exerted specific recognition and remarkable elimination against multiple CD5^+^ malignant cells even though the proportion of CD5 positivity on Jeko-1 cells was about 55% (Fig. 4a, Fig.S2c). No obvious difference in cytotoxicity was observed between 12 C CAR-NK cells and NT-NK cells after co-culture with control CD5^−^ cell line Raji (Fig. 4a). In addition, we constructed CD5^+^ Raji cells to further assess the specific cytotoxicity of 12 C CAR-NK cells. As a result, 12 C CAR-NK cells showed a remarkably greater cytotoxicity efficacy towards CD5^+^ Raji cells than NT-NK cells (Fig. 4a). Taken together, these results indicated that 12 C CAR-NK cells executed potent and specific cytotoxicity against CD5^+^ hematologic malignant cells.

In view of CD5 expression on the surface of normal T cells [36], the cytotoxicity of 12 C CAR-NK cells to normal T cells was evaluated. Autologous normal T cells (auto-T) and two donors’ normal T cells (T1 and T2) were used for coculture with 12 C CAR-NK cells and NT-NK cells. The results demonstrated that 12 C CAR-NK cells exerted cytotoxic ability against both auto-T cells and allogeneic normal T cells compared to NT-NK cells (Fig. 4b), which facilitated manufacturing “off-the-shelf” universal CD5 CAR-NK cell products with extremely diminished risk of GVHD even though a side effect towards normal T cells.

### 12 C CAR-NK cells cultured in the presence of engineered K562 feeder cells exert potent cytotoxicity against CD5+ hematologic malignant cells

**Fig. 5:**
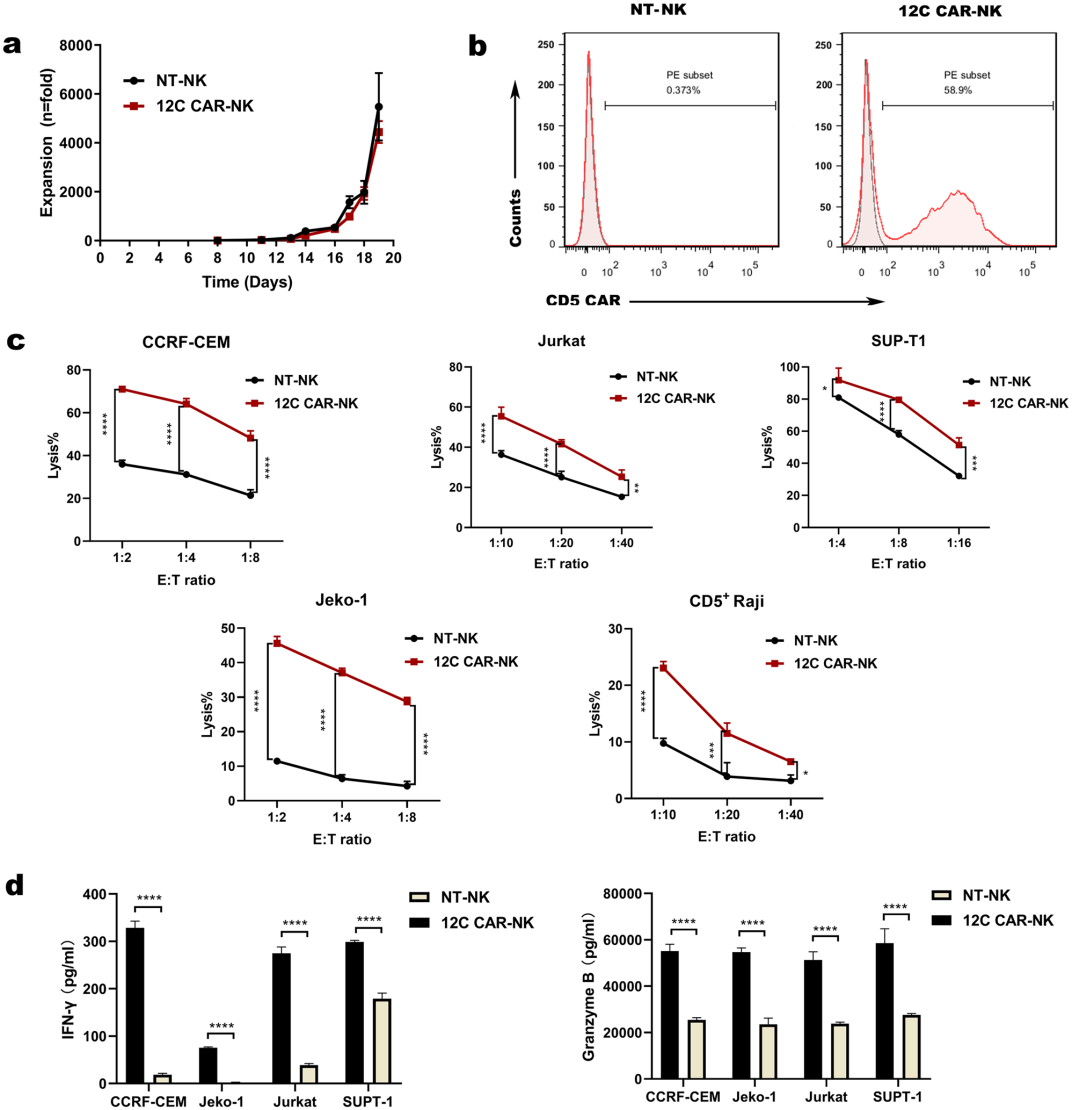
12 C CAR-NK cells with edited K562-based feeder cells exhibit potent cytotoxic activity against CD5^+^ hematologic malignant cells ex vivo. (**a**) Expansion of 12 C CAR-NK cells cultured with K562-based feeder cells expressing membrane-bound IL-21 and CD137 ligand and exogenous IL-2 (200 IU/mL) within 19 days. (**b**) Flow cytometry analysis of CD5 CAR expression on CAR-NK cells versus NT-NK cells on day 14. (**c**) Cytotoxicity of 12 C CAR-NK cells and NT-NK cells against CD5^+^ hematologic malignant cells was evaluated. Effector cells and target cells were cocultured overnight at the indicated E: T ratio (*n* = 3; two-way ANOVA; *p < 0.05, **p < 0.01, ***p < 0.001, ****p < 0.0001). (**d**) ELISA data showing the release of IFN-γ and Granzyme B by NT-NK and 12 C CAR-NK cells after co-incubated with different target cells overnight (*n* = 3; ****p < 0.0001). NT-NK: nontransduced NK; CD5^+^ Raji: Raji overexpressed membrane-proximal domain of CD5

To improve the proliferative ability of CAR-NK cells, we chose another NK expansion method that used K562-based feeder cells expressing membrane-bound IL-21 and CD137 ligand and exogenous IL-2 (200 IU/mL) to stimulate CAR-NK cells. On day 6, NK cells were transduced with a lentiviral vector carrying 12 C VHH, a CD28-derived hinge, a CD28 transmembrane and intracellular domain and a CD3ζ signaling domain, in combination with the human IL-15 gene to generate 12 C CAR-NK cells. We produced up to 4442 fold expansion of CAR-NK cells within 19 days of culture (Fig. 5a). Consistent with the culture method of cytokine stimulation aforementioned, the CD5 CAR expression on NK cells was 58.9% by flow cytometry on day 14 (Fig. 5b). To investigate whether the culture method of feeder cells could have influenced the cytotoxicity of 12 C CAR-NK cells against CD5^+^ hematologic malignant cells, we selected Jurkat, CCRF-CEM, SUPT-1, Jeko-1 and CD5^+^ Raji cells as target cells. After coculture with CD5^+^ target cells at different E: T ratios, 12 C CAR-NK cells exerted superior killing of CD5^+^ hematologic malignant cells compared to NT-NK cells (Fig. 5c). In addition, activated NK cells predominantly released INF-γ related to the cytotoxic activity of NK cells. Hence, when 12 C CAR-NK cells incubated with CCRF-CEM cells, INF-γ cytokine was detected, revealing that IFN-γ released by 12 C CAR-NK cells was significantly higher compared with that released by NT-NK cells, while a dramatic increase in Granzyme B released by 12 C CAR-NK cells was observed (Fig. 5d). In brief, 12 C CAR-NK cells cultured by modified K562 feeder cells exhibited excellent specificity and potent cytotoxicity against malignant T cells in vitro.

### IL-15△ CAR-NK cells attenuate the antitumor activity

**Fig. 6:**
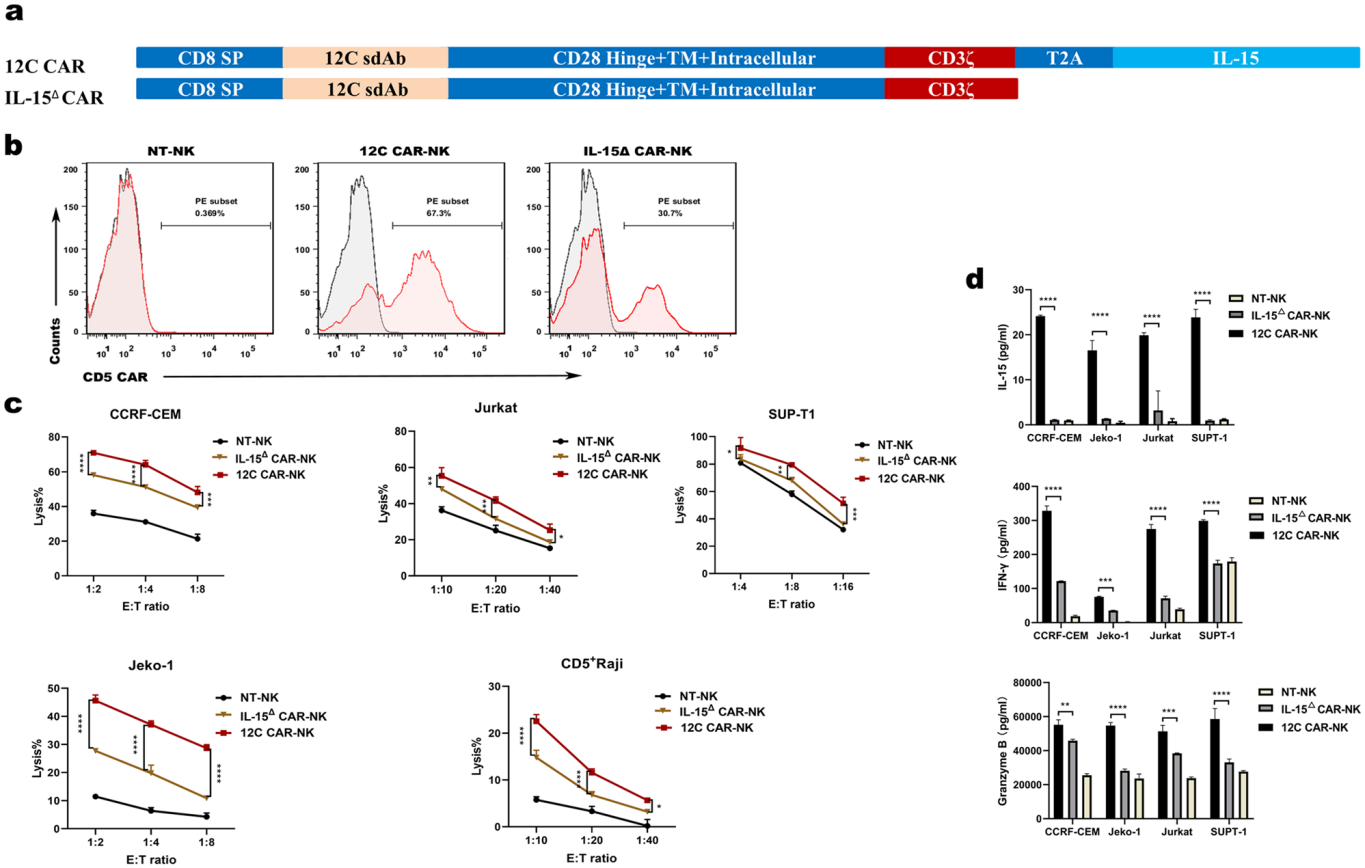
IL-15^△^ CAR-NK cells attenuate the capacity of eliminating tumor cells in vitro. (**a**) Schematic diagram of the two lentiviral CD5 CAR expression plasmids with IL-15 and without IL-15. (**b**) Flow cytometry analysis of CD5 CAR expression on CAR-NK cells versus NT-NK cells on day 14. (**c**) Cytotoxicity of 12 C CAR-NK, IL-15^△^ CAR-NK and NT-NK cells against CD5^+^ hematologic malignant cells was evaluated. (**d**) ELISA data showing the release of IL-15, IFN-γ and Granzyme B by NT-NK, 12 C CAR-NK and IL-15^△^ CAR-NK cells after co-incubated with target cells overnight (*n* = 3; *p < 0.05, **p < 0.01, ***p < 0.001, ****p < 0.0001). NT-NK: nontransduced NK; IL-15^△^: deficient in expression of IL-15

To test the role of IL-15 in the cytolytic activity of CAR-NK cells, we generated CAR-NK cells devoid of expression of IL-15 (IL-15^△^ CAR-NK). We performed in primary PB-derived NK cells followed by transduction of IL-15^△^ CAR composed of 12 C VHH, a CD28-derived hinge, a CD28 transmembrane and intracellular domain and a CD3ζ signaling domain (Fig. 6a). After expansion with modified K562 feeder cells, the transduction efficiencies of 12 C CAR-NK and IL-15^△^ CAR-NK cells on day 14 were 67.3% and 30.7%, respectively (Fig. 6b). However, specific killing by IL-15^△^ CAR-NK cells was significantly attenuated at different E: T ratios relative to 12 C CAR-NK cells (Fig. 6c) when cultured with CD5^+^ cell lines, CCRF-CEM, Jurkat, SUPT-1, Jeko-1 and CD5^+^ Raji in vitro, indicating that deletion of IL-15 compromised the lytic capability of CAR-NK cells. Then, we detected cytokine released by IL-15^△^ CAR-NK and 12 C CAR-NK cells in response to target cells. IL-15 was undetectable in supernatant collected from IL-15^△^ CAR-NK cells and NT-NK cells cultured with CCRF-CEM, Jurkat, SUPT-1 and Jeko-1 cells. On the contrary, a certain level of IL-15 was detected after 12 C CAR-NK cells killing multiple tumor cell lines (Fig. 6d). IFN-γ and Granzyme B released by IL-15^△^ CAR-NK cells were significantly lower than those released by 12 C CAR-NK (Fig. 6d). The results indicated that the lack of IL-15 attenuated the cytotoxicity of CAR-NK cells.

### 12 C CAR-NK cells exhibit superior antitumor activity in vivo

**Fig. 7:**
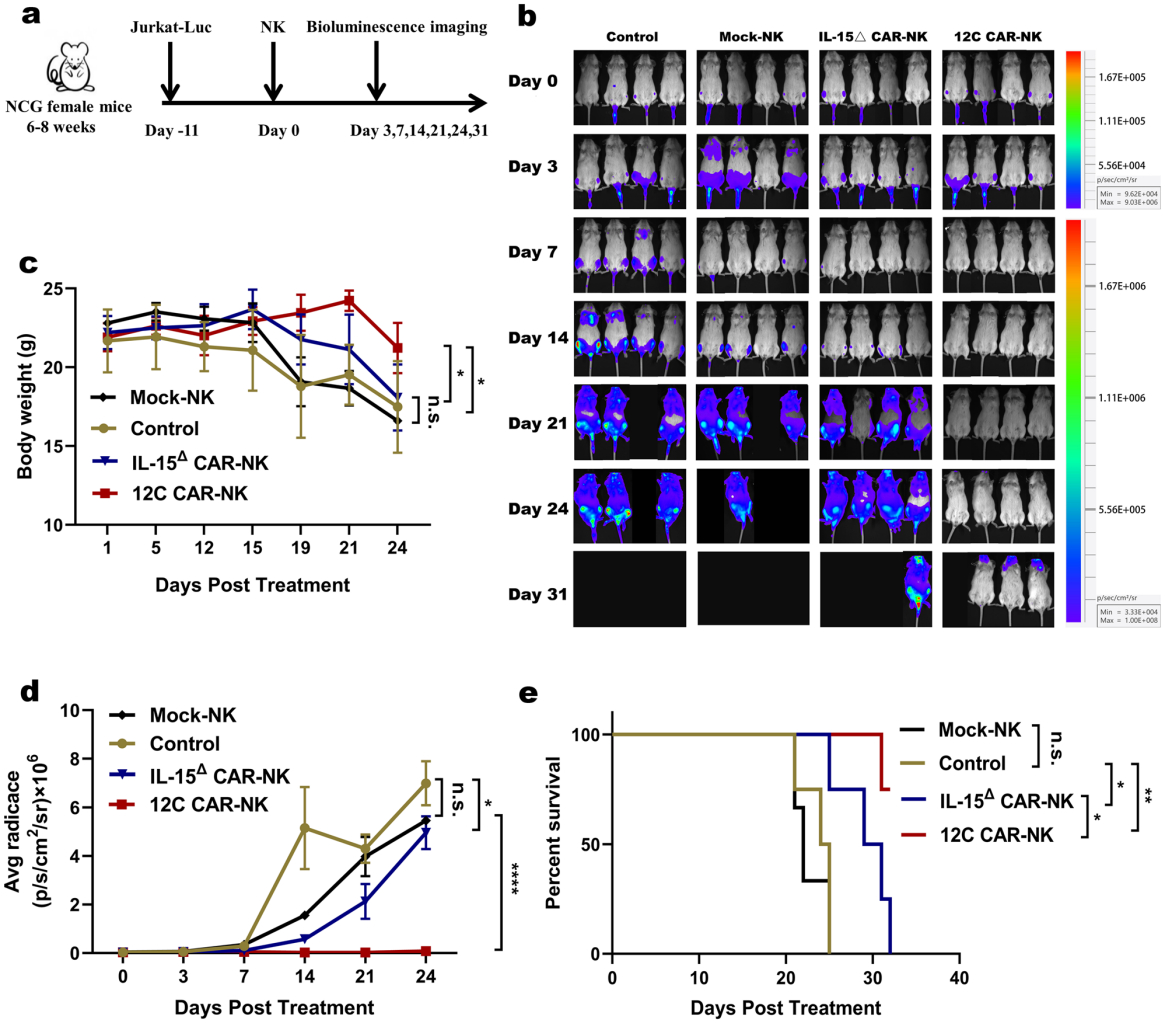
12 C CAR-NK cells exhibit superior antitumor activity in vivo. (**a**) Schematic diagram of the treatment process. Mice were intravenously injected with 3 × 10^6^ Jurkat-Luc cells. Eleven days after transplantation, mice were divided into four treatment groups (*n* = 4) according to the average radiance of the bioluminescent imaging: group freezing solution (control), group Mock-NK, group IL-15^△^ CAR-NK, and group 12 C CAR-NK. Mice were respectively intravenously administered with freezing solution or 5 × 10^6^ cells of either Mock-NK, IL-15^△^ CAR-NK, or 12 C CAR-NK cells. One mouse of Mock-NK group was not monitored tumor burden by bioluminescence images as failure of tumor-bearing on day 14 post-transplantation. (**b**) Tumor burden was monitored by bioluminescence images. (**c**) Body weight curve of each group at different days (*n* = 4; two-way ANOVA; *p < 0.05; n.s., no significance) (**d**) Statistical analysis of the bioluminescence intensity of each group at different days (*n* = 4; two-way ANOVA; *p < 0.05, ****p < 0.0001; n.s., no significance). (**e**) Kaplan–Meier survival curve (log-rank test). Mice treated with 12 C CAR-NK cells showed significantly increased survival compared with those of control, Mock-NK and IL-15^△^ CAR-NK groups. (*p < 0.05, **p < 0.01; n.s., no significance)

To further verify the antitumor activity of 12 C CAR-NK cells and the contribution of IL-15 to the efficacy in vivo, we established a mouse tumor model by tail intravenous injection of Jurkat-Luc cells. 3 × 10^6^ Jurkat-Luc cells were engrafted into six- to eight-week-old NCG mice and engraftment was corroborated by BLI after 11 days. Then, freezing solution or 5 × 10^6^ cells of either Mock-NK, IL-15^△^ CAR-NK, or 12 C CAR-NK cells were infused intravenously on day 11 after tumor engraftment and Jurkat-Luc cell growth was monitored by in vivo imaging as planned (Fig. 7a). In contrast to the mice in the control or Mock-NK cells groups, which revealed massive Jurkat-Luc cells, the tumor burden was alleviated in mice receiving CAR-NK cells, especially in mice by 12 C CAR-NK cells treatment (Fig. 7b). Twenty-four days after transplantation, the body weight of mice in the freezing solution and Mock-NK groups led to sharply decline, while that of the IL-15^△^ CAR-NK group slowly decreased. But, the body weight of mice treated with 12 C CAR-NK cells maintained stability (Fig. 7c). BLI showed a reduced tumor burden in the mice infused with 12 C CAR-NK cells compared with those infused with IL-15^△^ CAR-NK and Mock-NK cells on day 24 (Fig. 7d). The survival time of mice between control and Mock-NK groups was comparable. 12 C CAR-NK cells controlled tumor progression and prolonged survival better than the IL-15^△^ CAR-NK cells, emphasizing the crucial role of IL-15 to anti-tumor efficacy (Fig. 7e).

## Discussion

T cell malignancies tend to have dismal prognoses in the absence of curative treatments comparable to B cell malignancies. Treatment interventions of T-cell malignancies are with a significant unmet medical need. CAR-T cells against B-cell malignancies have been proven highly effective and broadly tolerable [37–39]. Whereas, broadening the success of CAR-T cells to tackle T-cell malignancies is problematic owing to the shared expression of surface antigens between normal and malignant T cells, leading to fratricide in CAR-transduced T cells. So far, modificated CAR-T cells targeting T-cell surface antigens such as CD3, CD4, CD5 and CD7 were deployed to circumvent the concerns [40–45]. Rasaiyaah et al. established a strategy via TALEN-mediated disruption of TCRαβ/CD3 complex followed by self-enrichment for CD3 CAR^+^ TCR^–^ CD3– T cells to overcome CAR-T cell fratricide [44]. Pinz and colleagues demonstrated that modified CD8^+^ T cells expressing CD4 CAR induced robust anti-tumor effects in vivo, and CAMPATH was used as a natural safety switch to prevent unwanted toxicities [43, 46]. Zhang et al. considered the issue of self-antigen-driven fratricide and addressed the drawback by anchoring CD7 in the ER and/or Golgi [47]. An alternative strategy deficient in the expression of CD7 with CRISPR/Case9 gene editing had been established to avoid fratricide [42, 48]. A recent report described that functionally blocking CD5 signaling heightened CD8^+^ T cell activation and antitumor activity [49]. Another study demonstrated that knockout of CD5 enhanced the cytotoxicity of CAR-T cells, and this correlated with boost of CAR-mediated activation and proliferation [50]. FHV_H_-derived CD5/CD7 bispecific CAR-T cells with CD5 and CD7 deletion had showed potent antitumor activity against T cell malignancies and tandem CARs could be conducive to mitigating tumor antigen escape [51]. So far, there have been a few of phase I studies (NCT03081910, NCT04689659, NCT04004637) in regard to the CAR-T cells targeting CD5 or CD7 to treat relapsed/refractory T-lymphocytic leukemia/lymphoma, but with tumor recurrence [52–54]. In recent years, CAR-NK cells have been a hopeful strategy to avoid fratricide. NK-92 cell line has been engineered to manufacture CAR NK-92 cells against CD3, CD4 and CD5 [9, 27, 55, 56], because NK cells derived from PB are challenging to expand and be transduced [57]. We chose to investigate CD5 antigen as CD5 is highly expressed in the majority of T-ALL and T cell lymphoma [58, 59], but also in a fraction of B cell tumor, such as CLL and MCL [60, 61]. The expression of CD5 has been considered to be a distinguishing feature of these diseases. Targeting CD5 with immunoconjugates linked to cytotoxic molecules had been used in clinical trials for cutaneous T-cell lymphoma [62]. Hence, it is feasible for CD5 CAR-NK cells against both T and partial B lymphoid malignancies. In addition, CD5 is not expressed on the surface of hematopoietic stem cells and NK cells [63]. When targeting the antigen, hematopoietic stem cells and NK cells will not be affected to avoid impairing cell differentiation and the function of NK cells. The ideal target is the antigen expressed in T cells, which is diminished without compromising immune function. CD5 blockade with anti-CD5 monoclonal antibody or CD5 knockout could be therapeutically beneficial to enhance T cell-mediated anti-tumor immunity [49, 64]. Thus, CD5 can be used as an attractive target for CAR-NK immunotherapy to treat both T and partial B-cell malignancies.

In this study, we developed “off-the-shelf” CAR-NK therapy targeting CD5-D3 derived from VHH against T-cell malignancies, permitting the use of allogeneic donor NK cells without the risk of GVHD. Traditional antigen recognition module of CAR is usually a murine single-chain variable fragment (scFv) composing of heavy- and light-chain variable connected by a flexible linker, linked to cytoplasmic signaling domain. Compared with scFv, VHH possesses apparent advantages in constructing CARs. First, the similarity of the VHH sequence to human immunoglobulin V regions offers an important advantage in low-immunogenicity. Second, only single-domain antibody fragments render the structural design of CARs more simplified. Third, the small size of VHH, approximately 15 kDa, provides a potential steric which benefits for accessing cryptic antigenic epitopes different from those recognized by scFvs [65]. Finally, the strict monomeric behavior of VHH has a role in retaining the protein stability and binding affinities [66], and yielding a highly modular platform without requirement for extensive reformatting. Several clinical studies of anti-B-cell maturation antigen (BCMA) VHH CAR-T or CAR-NK therapies [67–69] showed similar clinical efficacy for scFv-based BCMA CAR-T therapy [70], demonstrating favorable perspectives for immunotherapy. Membrane-proximal region of antigen molecule may be responsible for special ability to cause stronger antitumor immunity, which had been applied to anti-CD22 CAR-T targeting the most proximal Ig domain (d7) [19] and anti-Mesothelin CAR-T targeting region III [20], as well as HIV vaccine design [71]. Those results showed that the membrane-proximal region was be considered as the pivotal structure to provoke stronger immune response. Therefore, we screened the VHHs specially binding to membrane-proximal domain of CD5, followed by constructing CD5 CAR-NK cells in order to expect stronger antitumor response. The superior properties of VHH make them ideal building blocks for above special CAR constructs.

Sufficient cell expansion and large amount of cells are the crucial issues for clinical application of NK cells. In a phase I clinical trial, 12 patients with refractory acute myeloid leukemia were administrated with expanded high dose of NK-cells (10^6^-10^7^/kg/dose) using membrane-bound IL-21 and CD137 ligand expressed on K562. Seven patients (58.3%) achieved a complete remission without adverse effects related to NK-cell infusions [72]. In the study, the expansion of 12 C CAR-NK cells with K562 feeder system was over 4000 fold, obviously superior to that of CAR-NK cells with cytokine stimulation. The resistance of PB-derived NK cells to transduction is a virtual hurdle in the successful modification of CAR-NK cells. BaEVRLess promoting viral entry had been developed, achieved a transduction efficiency of 38.3%±23.8% and 58.4%±7.8%. The transduction was performed before second expansion of NK cells [73]. There are few reports on nanobody-derived CAR-NK cells. Previous research showed BCMA CAR expression of PB-derived NK cells was 38.4% and VHH directed BCMA CAR-NK cells exhibited remarkable specific killing ability [68]. In the study, the average % CAR transduction was 64.2% from three healthy donors with K562 feeder system (data not shown), this high transduction facilitates the clinical application of nanobody-derived CAR-NK cell derived from PB.

The limited lifespan and poor in vivo persistence of mature NK cells pose significant challenges for achieving sustained clinical responses in adoptive therapy. However, we addressed this issue by incorporating the gene encoding IL-15 into the CAR construct, resulting in improved proliferation, persistence, and cytotoxicity of NK cells. The feasibility of this strategy had been verified in a clinical trial [28]. In the study, 12 C CAR-NK cells demonstrated superior antitumor activity both in vitro and in vivo. To further emphasize the role of IL-15, we also generated 12 C CAR-transduced NK cells lacking IL-15, which showed attenuated antitumor response. This highlights the substantial impact of IL-15 on achieving effective and durable antitumor responses. This modification has provided a promising solution to overcome the limitations of NK cell persistence and has demonstrated its efficacy in enhancing antitumor activity. The inclusion of IL-15 in the CAR construct holds great potential for improving the outcomes of adoptive therapy. In addition, The signaling domain of CAR-NK is mainly based on structure of CAR-T cells. CD28, an important costimulatory molecule for T cell activation [74], is expressed in human fetal NK cells and a number of NK cell lines [75, 76]. CD28 ligation in NK cells strengthens the activity of NK cells by phosphorylating ERK2 [77]. NK cells transduced with a CAR incorporating CD28 showed stronger cytotoxicity than 2B4 as intracellular domain [68]. Hence, it demonstrates the feasibility of CD28 as costimulatory molecule in NK cells.

The cytokines predominantly produced by NK cells are IFN-γ and GM-CSF. In the study, 12 C CAR-NK cells were associated with inferior IFN-γ released, which mitigating the risk of the concerns. Moreover, rapid weight loss and early deaths in the animal model were not observed. The absence of overt toxicity supports the potential development of our approach. In addition, 12 C CAR-NK cells showed cell lysis properties towards normal autologous and allogeneic T cells due to CD5 expression in normal T cells, indicating that T-cell immunodeficiency would be induced by 12 C CAR-NK cells targeting T cell malignancies. While lymphopenia is generally undesirable, a further strategy of bridging allogeneic HSCT after complete remission to reconstitute patients’ immune systems is to abrogate the risk of eradicating host T lymphocytes. In other words, T cell aplasia caused by CD5 CARs targeting T cell malignancies can be considered as a novel conditioning regimen.

In conclusion, this study presents the first report of targeting CD5-D3 VHH combining armored CAR in PB-derived NK cells. We screened the membrane-proximal domain VHH targeting CD5 associating with engineered human NK cells, followed by lentiviral transduction with a fourth generation CD5-CAR resulting in 12 C CAR-NK cells that exhibited excellent cell lysis properties against T cell malignancy in vitro and in vivo. The efficacy of adoptive transfer of CD5 CAR-NK cells warrants further efforts to clinical application for patients with T-cell hematologic malignancies in a clinical trial setting.

## Electronic supplementary material

Below is the link to the electronic supplementary material.

Supplementary Material 1: **Supplemental Fig. 1: characterization of anti-CD5 VHH**. (a) SDS-PAGE analysis of purified VHHs. (b) Amino acid sequence alignment of domain 3 between mouse CD5 protein (top panel) and human CD5 protein (bottom panel). The same amino acid sequences were depicted in middle panel. (c) VHH-Fc recognition specificity for CD5 protein of different species. Two VHHs recognized human CD5-FL and CD5-Trunc proteins with high-binding ability rather than mouse CD5 protein, as determined by ELISA assay (*n* = 2). Antibody concentration was 10 µg/mL. CD5-FL: Full-length CD5; CD5-Trunc: Membrane-proximal domain of CD5; VHH: Variable domain of heavy chain of heavy-chain. **Supplemental Fig. 2: the surface expression of CD5 in different tumor cell lines**. (a) Flow cytometry analysis of CD5 expression on the cell surface of T cell leukemia cell lines, CCRF-CEM and Jurkat. (b) Flow cytometry analysis of surface expression of CD5 on the cell surface of CD5-Trunc overexpressed Raji. (c) Flow cytometry analysis of CD5 expression on the cell surface SUP-T1 in high level and on Jeko-1 in low level, CD5-negative Raji cell line was as negative control. CD5-Trunc: Membrane-proximal domain of CD5

## Data Availability

The data-sets used and/or analyzed during the current study are available from the corresponding author on reasonable request.

## Abbreviations

T-ALL: T-cell acute lymphoblastic leukemia
HSCT: Hematopoietic stem cell transplantation
MCL: Mantle cell lymphoma
CLL: Chronic lymphocytic leukemia
TCR: T-cell receptor
CAR: Chimeric antigen receptor
GVHD: Graft-versus-host-disease
NK: Nature killer
MHC: Major histocompatibility complex
CRS: Cytokine release syndrome
ORR: Overall response rate
SRCR: Scavenger receptor cysteine-rich
PBMCs: Peripheral blood mononuclear cells
PB: Peripheral blood
LV: Lentivirus
PBLs: Peripheral blood lymphocytes
RT-PCR: Reverse transcription polymerase chain reaction
CDR: Complementarity determining region
SDS-PAGE: Sodium dodecyl sulfate-polyacrylamide gel electrophoresis
EC50: Concentration for 50% of maximal effect
ER: Endoplasmic reticulum
MOI: Multiplicities of infection
SD: Standard deviation
ScFv: Single-chain variable fragment
BCMA: B-cell maturation antigen
