# Supplementary material for: Targeting CD5 chimeric antigen receptor-engineered natural killer cells against T-cell malignancies
Source: Exp Hematol Oncol. 2024 Oct 26;13:104. doi: 10.1186/s40164-024-00577-5 (PMC11515150; doi:10.1186/s40164-024-00577-5)

**Supplemental Figure 1: Characterization of anti-CD5 VHH.** (a) SDS-PAGE analysis of purified VHHs. (b) Amino acid sequence alignment of domain 3 between mouse CD5 protein (top panel) and human CD5 protein (bottom panel). The same amino acid sequences were depicted in middle panel. (c) VHH-Fc recognition specificity for CD5 protein of different species. Two VHHs recognized human CD5-FL and CD5-Trunc proteins with high-binding ability rather than mouse CD5 protein, as determined by ELISA assay (n=2). Antibody concentration was 10µg/mL. CD5-FL: Full-length CD5; CD5-Trunc: Membrane-proximal domain of CD5; VHH: Variable domain of heavy chain of heavy-chain.


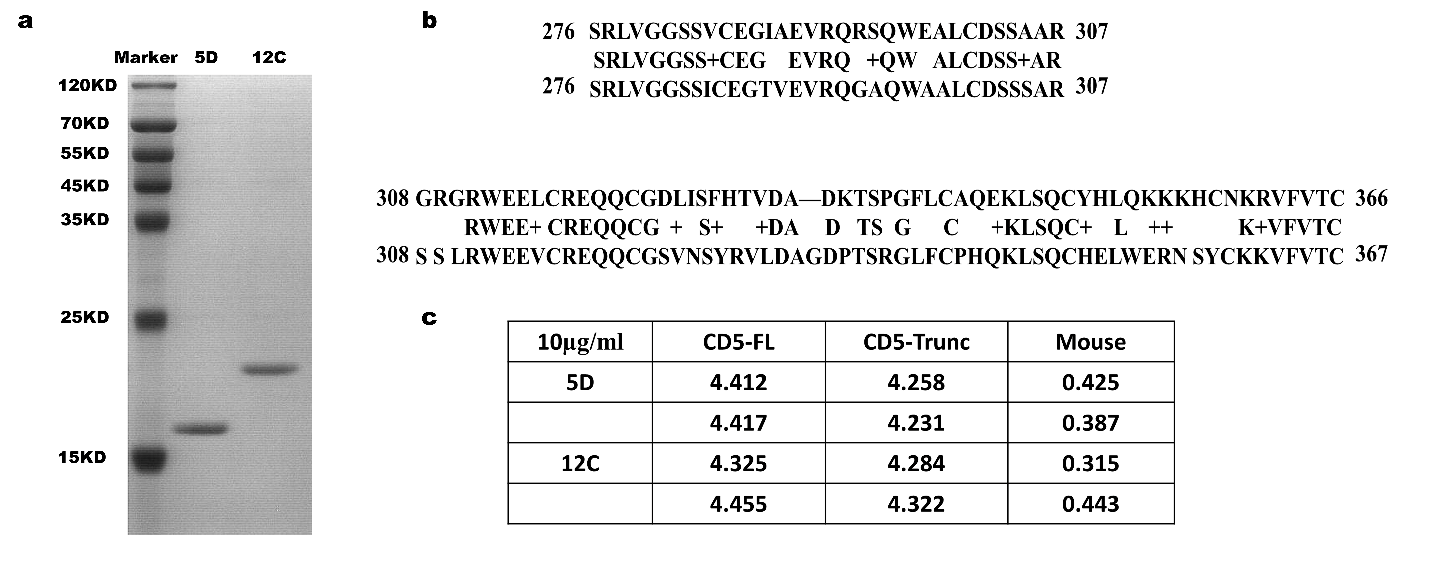


**Supplemental Figure 2: The surface expression of CD5 in different tumor cell lines.** (a) Flow cytometry analysis of CD5 expression on the cell surface of T cell leukemia cell lines, CCRF-CEM and Jurkat. (b) Flow cytometry analysis of surface expression of CD5 on the cell surface of CD5-Trunc overexpressed Raji. (c) Flow cytometry analysis of CD5 expression on the cell surface SUP-T1 in high level and on Jeko-1 in low level, CD5-negative Raji cell line was as negative control. CD5-Trunc: Membrane-proximal domain of CD5.


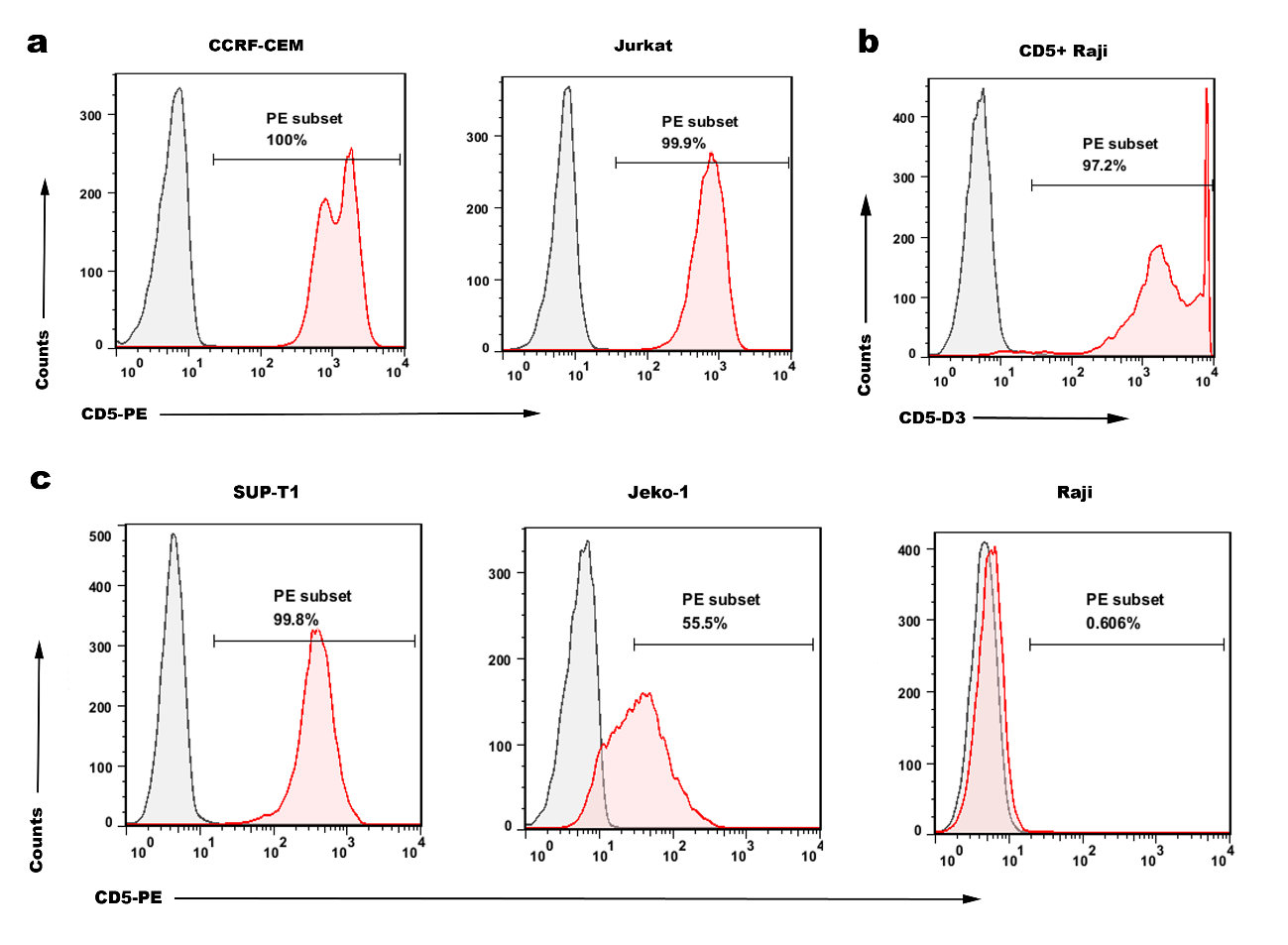

Supplement: Supplementary file 1 — Supplementary Material 1: Supplemental Fig. 1: characterization of anti-CD5 VHH. (a) SDS-PAGE analysis of purified VHHs. (b) Amino acid sequence alignment of domain 3 between mouse CD5 protein (top panel) and human CD5 protein (bottom panel). The same amino acid sequences were depicted in middle panel. (c) VHH-Fc recognition specificity for CD5 protein of different species. Two VHHs recognized human CD5-FL and CD5-Trunc proteins with high-binding ability rather than mouse CD5 protein, as determined by ELISA assay (n = 2). Antibody concentration was 10 µg/mL. CD5-FL: Full-length CD5; CD5-Trunc: Membrane-proximal domain of CD5; VHH: Variable domain of heavy chain of heavy-chain. Supplemental Fig. 2: the surface expression of CD5 in different tumor cell lines. (a) Flow cytometry analysis of CD5 expression on the cell surface of T cell leukemia cell lines, CCRF-CEM and Jurkat. (b) Flow cytometry analysis of surface expression of CD5 on the cell surface of CD5-Trunc overexpressed Raji. (c) Flow cytometry analysis of CD5 expression on the cell surface SUP-T1 in high level and on Jeko-1 in low level, CD5-negative Raji cell line was as negative control. CD5-Trunc: Membrane-proximal domain of CD5 [file 40164_2024_577_MOESM1_ESM.docx]
